# Feature Extraction Using a Residual Deep Convolutional Neural Network (ResNet-152) and Optimized Feature Dimension Reduction for MRI Brain Tumor Classification

**DOI:** 10.3390/diagnostics13040668

**Published:** 2023-02-10

**Authors:** Suganya Athisayamani, Robert Singh Antonyswamy, Velliangiri Sarveshwaran, Meshari Almeshari, Yasser Alzamil, Vinayakumar Ravi

**Affiliations:** 1 School of Computing, Sastra Deemed to be University, Thanjavur 613401, India; 2 Department of Computational Intelligence, School of Computing, SRM Institute of Science and Technology, Kattankulathur, Chennai 603203, India; 3 Department of Diagnostic Radiology, College of Applied Medical Sciences, University of Ha’il, Ha’il 55476, Saudi Arabia; 4 Center for Artificial Intelligence, Prince Mohammad Bin Fahd University, Khobar 34754, Saudi Arabia

**Keywords:** spatial gray level dependence matrix, Canny algorithm, modified chimp optimization algorithm, softmax classifier, deep convolutional neural network

## Abstract

One of the top causes of mortality in people globally is a brain tumor. Today, biopsy is regarded as the cornerstone of cancer diagnosis. However, it faces difficulties, including low sensitivity, hazards during biopsy treatment, and a protracted waiting period for findings. In this context, developing non-invasive and computational methods for identifying and treating brain cancers is crucial. The classification of tumors obtained from an MRI is crucial for making a variety of medical diagnoses. However, MRI analysis typically requires much time. The primary challenge is that the tissues of the brain are comparable. Numerous scientists have created new techniques for identifying and categorizing cancers. However, due to their limitations, the majority of them eventually fail. In that context, this work presents a novel way of classifying multiple types of brain tumors. This work also introduces a segmentation algorithm known as Canny Mayfly. Enhanced chimpanzee optimization algorithm (EChOA) is used to select the features by minimizing the dimension of the retrieved features. ResNet-152 and the softmax classifier are then used to perform the feature classification process. Python is used to carry out the proposed method on the Figshare dataset. The accuracy, specificity, and sensitivity of the proposed cancer classification system are just a few of the characteristics that are used to evaluate its overall performance. According to the final evaluation results, our proposed strategy outperformed, with an accuracy of 98.85%.

## 1. Introduction

Lesions that are deemed too small to target properly and safely, as well as patients who are coagulopathic or otherwise unable to safely sustain intravenous sedation or general anesthesia, are the absolute contraindications to brain biopsy. Imaging tests include X-rays, CT scans [1], and MRIs. MRIs are used for the purpose of creating precise, computer-generated images of the body by employing strong magnets and radio waves. There is a small, tunnel-like aperture on a typical MRI machine, resembling a donut. X-rays and other kinds of radiation are not used in an MRI. To check for issues with the reproductive system, doctors frequently utilize this method. Even for expectant mothers, an MRI is typically safe. Additionally, doctors utilize it to photograph the brain, spinal column, abdomen, and chest, including the breast.

A mass or collection of aberrant brain cells is known as a brain tumor. The brain is protected by the very strong skull. Any growth within such a constrained area can lead to issues. Malignant (cancerous) or noncancerous (benign) brain tumors are possible. The pressure inside your skull may rise as benign or cancerous tumors enlarge. This has the potential to be fatal and can result in brain damage.

One of the imaging methods currently in use is magnetic resonance imaging (MRI). In the present circumstances, MRI is a beneficial source of medical data. People’s lifestyles are changing, and that is putting them at risk of a variety of health concerns. One of these issues is cancer, the detection and diagnosis of which are generally regarded as laborious tasks. An MRI provides a significant amount of information regarding the patient. Detecting cancer is one area where it can be of great assistance.

Consequently, magnetic resonance imaging is a significant data source that is coming to be utilized extensively in various medical applications [2], particularly given the phenomenal increase in brain tumors (BT). It is of the utmost importance to locate the most effective treatment for early brain tumors using MRI [3]. The automation of the classification process helps radiologists with particular brain MRI classification and decreasing the interference tumors cause. Finding and identifying the type of brain tumor in an MRI image is an important and fundamental step in biomedical image processing [4]. In image segmentation, the goal is to cluster the image’s pixels into constrained groups and then apply a stimulating marker to each of those groupings individually. When it comes to examining and analyzing therapeutic images, the segmentation of the images plays a significant role. The segmentation of the images focuses on selecting a ROI (region of interest) or recognizing objects [5,6].

Currently, there are numerous subtypes of brain tumors to identify. Generally, these can be broken down into benign and malignant. Benign tumors are regarded as low-grade tumors, and they do not contribute to the development of cancer [7,8]. That said, although they do not pose a significant threat to the patient, diagnosing and treating benign tumors is still essential. The inability of the benign tumor to metastasize to other parts of the body is the primary distinguishing feature of this type of tumor. If the tumor is cancerous, it can simultaneously cause the body to experience a wide range of debilitating symptoms. Tumors of the malignant sort are what we mean when we talk about cancer. They can grow rapidly and ruin the health of the tissues they make contact with. If a tumor of this kind is found in the brain, medical professionals refer to it as PMT (primary malignant tumor). Secondary tumors can form in any brain location and quickly spread to other brain parts [9]. Meningioma, glioma, and pituitary tumor cells are kinds of tumor cells that can develop in the brain [10,11]. The detection of tumors automatically is a promising area of research, and significant progress has already been made. Most researchers use a segmentation-based methodology to approach the tumor detection model.

Clustering, thresholding, edge detection, and other similar methods are among the existing segmentation-based algorithms [12]. The thresholding method is the most effective and straightforward approach to tumor segmentation. The name comes from the fact that it operates primarily based on a threshold set [13]. Clustering is the process of grouping pixels into distinct groups that can then be segmented. This suggests that each cluster has a set of pixel values that indicate shared characteristics with the other cluster members. The segmentation process aims to simplify the image into a format that is easy to comprehend. Edge detection is one of the essential tasks in image processing [14,15]. Since the number of people diagnosed with brain tumors is climbing at an alarming rate, researchers are focused on developing and deploying the most promising technologies for brain tumor classification [16]. The first method of automatically classifying data is known as machine learning. The expertise of the specialists is crucial to the success of the classification process, which is based on the degraded characteristics [17]. Despite the drawbacks of machine learning approaches, only a few studies achieved a low classification accuracy based on the degraded features of MR images for tumor classification [18,19]. These features included the tumor’s shape, invariant texture, rotation, and intensity. In recent years, deep learning (DL) techniques [20], such as deep neural networks, have been utilized to classify images through self-learning without the need for human feature mining [21]. This has been conducted in response to the issue described above.

Convolutional neural networks are the most valuable DL strategy for addressing complicated problems in various applications such as localization, segmentation, recognition, and classification [22]. Other DL methods include deep learning and reinforcement learning. A deep convolutional neural network (DCNN) is currently the most successful method for image classification. Researchers have discovered that employing medical images yields superior results, and the ability of deep learning models to train on these images presents the greatest opportunity for applying such models. That line of development may overcome existing brain tumor detection drawbacks, provide a higher classification accuracy, and allow for superior analysis of visual features.

Recent research has shown that detecting tumors in biomedical images is an essential procedure that clinical specialists must carry out to keep up with the most recent developments in medical analysis. A brain tumor is defined as the formation of abnormal cells in the brain, which can result in an excessive amount of cell division and can potentially be lethal. In the first diagnostic method, medical image analysis employs magnetic resonance imaging (MRI) and magnetic resonance spectroscopy to locate brain tumors. A qualified medical professional looks at several types of medical imaging to identify the likely locations of malignant tumors and the signs that they are there. This method of diagnosing tumors does not involve any invasive surgery. The primary purpose of imaging systems is to capture medical images to diagnose malignant diseases. The obtained pictures are processed using a series of algorithms based on software to differentiate the potentially malignant tumor location from the healthy tissue surrounding it. In the field of medical imaging, segmentation is considered to be one of the most significant activities. This operation can be accomplished manually by an expert with a high level of accuracy, but it is a slow process. Since the work of radiologists is so laborious and time-consuming, there is a pressing demand for a method of segmentation that is semi-automated. This could make up for the drawbacks of the automatic segmentation procedure while simultaneously giving the radiologist control over the segmentation process.

The contaminated area is isolated from the rest of the image thanks to the segmentation of the image. When assessing the size and location of a tumor, it is helpful to use a precise segmentation method. This makes treatment planning much more straightforward. To accomplish this, a physician with the necessary training must create the first scenario or provide training data for the classification. Numerous research studies have been conducted to classify various cancers based on the information gleaned from their associated medical imaging.

The following provides a synopsis of the work’s most significant contributions.

- An efficient new automatic classification system has been developed based on using a deep learning network to categorize the various types of brain cancers.
- Researchers have been able to recognize malignancies such as meningioma, glioma, and pituitary tumors using the newly developed deep learning network structure of ResNet-152 as a pre-trained model in the deep convolutional neural network.
- The use of a new adaptive Canny Mayfly algorithm for edge detection has been implemented.
- The substantial training dataset has been improved with the help of a method called data augmentation.
- The redundant features have been removed with the help of a modified version of the chimp optimization algorithm, increasing the classification accuracy.

A review of the works associated with our proposed algorithm can be found in Section 2, while the proposed approach can be found in Section 3. Results for the proposed methodology are then presented in Section 4, and Section 5 concludes the paper. 

## 2. Related Works

There are numerous existing works on MRI image segmentation and classification [23]. Jyothi et al. [24] summarized the various MRI image segmentation and classification methods that have been published in last two decades. Using an improved version of the edge detection method, Ahmed et al. [25] were able to locate malignancies in the human brain using magnetic resonance imaging (MRI). The researchers presented the genetic algorithm (GA), an algorithm that can detect edges. The MRI scan of the patient is used to locate the margins of the brain tumor. The balance contrast enhancement (BCE) technique, which gives superior image characteristics, is used to enhance picture features. This results in improved medical image features. The GA-based edge detection method with the appropriate training dataset was offered as a solution to identify the fine edges. The effectiveness of the proposed method was evaluated in comparison to those of several existing methods. 

Jaspin and Suganthi [26] carried out brain tumor segmentation and morphological edge detection of MR images, which depend on regional growth (RG), and the performance was evaluated using the FCM approach (fuzzy C-means). All three methodologies—RG, ROI, and morphological operations—were combined in this investigation. The solution proposed was preprocessing to get rid of noise, area growth based on FCM, and edge identification by morphological operations to improve the image. The FCM technique was used to segment the tumor after morphological operations such as erosion and dilatation were applied to the tissue sample.

Debanjan et al. [27] proposed that better automatic segmentation and recognition of brain MRIs may be achieved based on the QIS network. Using a combination of self-supervised training and a novel QIS-Net tailored to brain MRI segmentation was the key to successfully overcoming the challenges presented by CNN models. The proposed QIS-Net structure, denoted by the notation q-bits, is composed of three layers of quantum neurons. The beginning layer and the layer in the middle were linked in the QIS network topologies by transmitting quantum states in both directions. Without taking training into account, the image pixel intensities between these two layers were able to self-organize here. 

Zhang et al. [28] were tasked with performing brain tumor segmentation for a multi-modality MRI and chose to use TIU-Nets. This study proposed TIU-Nets as a method for segmenting brain gliomas. The segmentation processes, categorized into multiclass (MU-Net) and binary class (BU-Net), are referred to as TIU-Nets. The multi-resolution capabilities presented here come from BU-Net and are utilized by MU-Net. It was suggested that BU-Net should be utilized to forecast the segmentation soft-mask used. A candidate glioma region was produced to direct the multiclass segmentation of MU-Net using the weighted technique. This was accomplished by removing the backgrounds that did not include gliomas. The boundary data of the glioma structure were improvised in MU-Net by using the edge branch as a source of information.

Singh et al. [29] suggested a technique to find brain tumors. This work used a fully connected pyramid pooling network (FCPPN) to segment the tumor, to find the particular location or the type of tumor sought to classify. a multi-tier convolutional neural network with channel preference is the name of the classification method (MTCNNCP). After the tumor is classified, predicting survival may be challenging. Multi-tier Zernike (MTZR) is the name of the prediction, which is carried out using synthetic choices. The geometric distance is calculated to determine the tumor’s severity. In order to implement denoising, the same authors [30] suggested a novel adaptive diffusivity function that is defined by partial differential equations. The diffusivity function uses a gradient, Laplacian, and adaptive threshold to improve pictures of brain tumors while maintaining image detail. The improved image is fed into an improved multi-kernel fuzzy c-means (MKFCM) algorithm for image segmentation. Finally, it makes a distinction between tumor and normal tissue.

## 3. Motivation

The incidence rate of brain tumors is rapidly rising and they rank as the most dangerous disease in the modern world. There are a significant number of factors that contribute to this circumstance, and one of those factors is a shift in lifestyle. The condition may result in death, and there has been a rapid expansion of malignant tumors. However, if a tumor is discovered in the early phases, that risk can be reduced to a certain degree. Recognizing and categorizing different types of tumors is believed to be a laborious task. Many researchers are concentrating on this area, seeking to create more advanced technology. The primary objective of detection is to categorize tumors into one of two categories: normal or abnormal. The most recent study found over 120 different types of brain tumors; these include pituitary, meningioma, and glioma, amongst others. Different types of tumors can be distinguished based on various features, including their size, location, shape, and intensity. However, the meningioma and the pituitary have a similar texture, and their intensities are consistent, meaning they can be difficult to effectively distinguish. To address such concerns, the authors of this study propose a novel method for the multiclass categorization of brain tumors that is founded on deep learning technology.

## 4. The Proposed Tumor Classification Method

**Figure 1:**
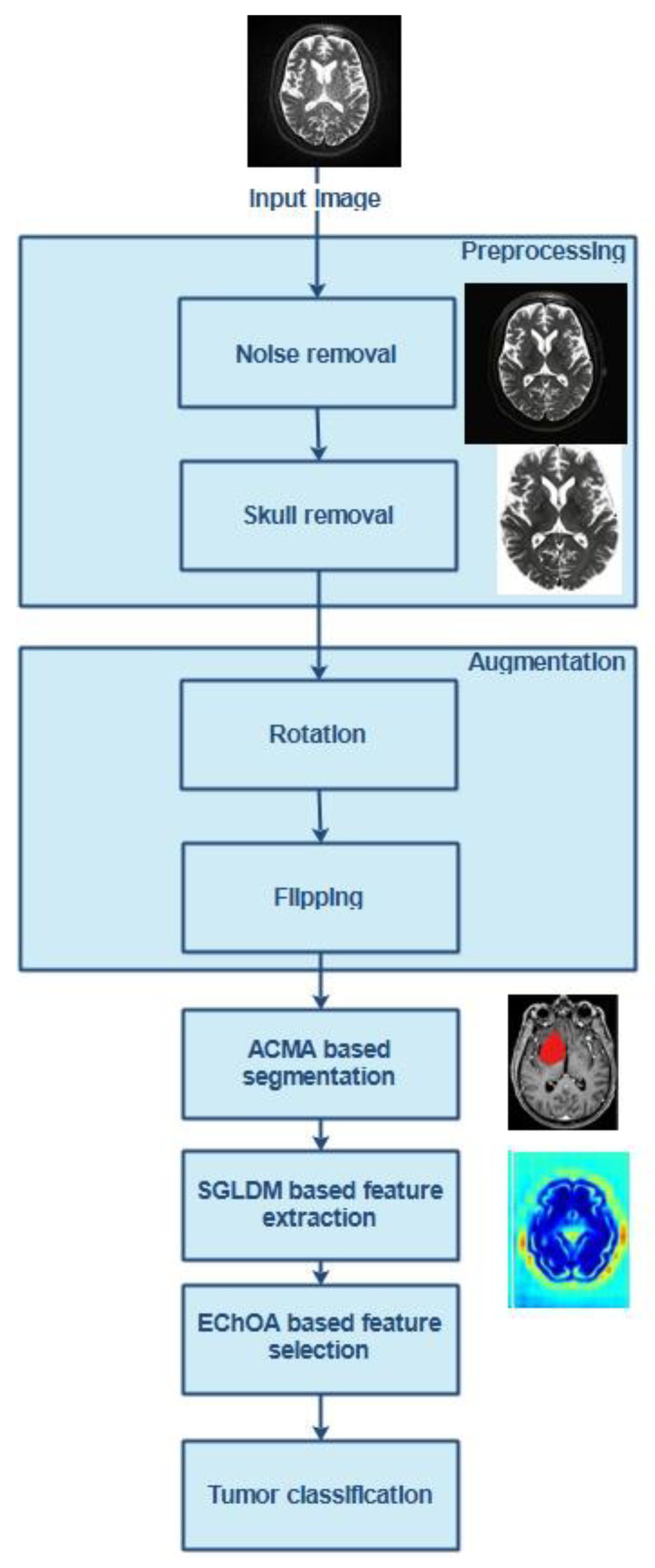
Overview of the tumor classification method.

It is challenging to categorize brain tumors into one of several available categories because each tumor has unique characteristics. Nowadays, deep neural networks for medical image categorization are primarily utilized in practice to assist neurologists. Overfitting is the deep network’s most significant drawback, along with gradient difficulties. To solve these issues, the authors of this work devised a novel automatic categorization system for dividing tumors into their respective categories. Figure 1 depicts the workflow that would be used for the proposed solution.

### 4.1. Materials and Methods

#### 4.1.1. Overview of the Proposed Tumor Classification Method

The process begins with preprocessing and continues with data augmentation, segmentation, feature extraction, selection, and classification. An adaptive filtering method is initially utilized to remove the noise. With the use of morphological surgeries known as erosion and dilation, the skull visible in the brain MRI image is also removed. After completing any necessary preprocessing steps, the data augmentation strategy consisting of rotation and flipping is applied to the training dataset to enhance the performance and provide CNNs with a larger input space. In addition, the augmentation procedure helps lessen the overfitting problem during training, which, in turn, helps increase the performance in generalization tasks. During the segmentation process, adaptive Canny Mayfly algorithm (ACMA)-based edge detection is utilized to identify the edges of the brain pictures being examined. Identification of edges will ensure the fine tumor boundary is outlined. After the segmentation is complete, SGLDM is used to extract the features (spatial gray level dependence matrix). Afterward, a tweaked version of the chimp optimization technique is used to choose the features (EChOA). In the final step of the classification process, the chosen features are used as input. During the classification process, a widely utilized deep network of residual networks known as ResNet-152 is utilized as the pre-trained network in DCNN. This removes the need for concern regarding the gradient problem. When it comes to the multiclass categorization of brain tumors, another softmax classifier is utilized.

#### 4.1.2. Pre-Processing

**Figure 2:**
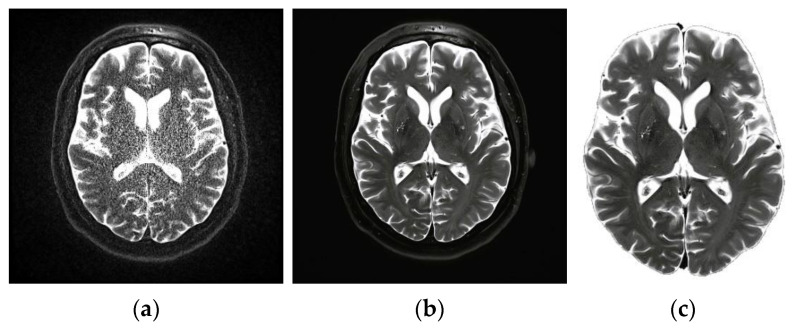
(**a**) Noisy MRI image, (**b**) filtered image, (**c**) and output of skull elimination.

It is generally agreed that the first step is the most crucial of all the steps. This procedure was primarily geared toward enhancing the image quality by removing any noise or other unnecessary components already present in the image. Eliminating background noise is challenging, especially in therapeutic and diagnostic settings. The preprocessing step in magnetic resonance imaging (MRI) can be difficult due to some issues, including an inhomogeneous magnetic field, patient movement, and external noise. As a result, this work suggests using an adaptable filtering method. The median filter [31] retains the relevant information that is already there in the image while simultaneously reducing the amount of noise. When using this method, each pixel in the image is evaluated concerning the pixels surrounding it, and the results are categorized as noise. After that, the value of the median pixel relative to its neighbors is substituted for these pixels. The smoothing of images is a fundamental practical module that improves image quality by reducing the amount of noise in the picture. Figure 2a represents a noisy MRI image, and Figure 2b represents the filtered image. The filtering is an optional step, where based on the amount of noise, a filtering decision will be made. Following the filtering step, the presence of the skull should be eliminated from the brain’s MRI by using erosion and dilation procedures. The erosion technique erases both the foreground and background of the skull. The existence of a false background leads to the development of some aberrations in the tissues of the brain. A procedure known as dilation is used on the tissue that has been stretched out of its standard shape. Generally, this method is quite effective at removing the skull from the MRI. The result of this step is shown in Figure 2c.

#### 4.1.3. Data Augmentation

Following the preprocessing step, the training dataset is then improved using a data augmentation strategy that involves rotating and flipping the data. As a direct consequence, the input space of CNN will be significantly expanded. In addition, the generalization performance is improved by this augmentation method, which also helps lessen the overfitting problem that occurs during training [32]. The result of flipping something is to produce a mirror reflection about some axis of your choosing. It is possible to say that the brain is anatomically symmetrical along numerous axes due to the presence of two hemispheres in the axial plane of the organ. The left hemisphere is replaced with the right hemisphere and vice versa when the object is flipped along the horizontal axis. This method’s identification process is quite effective when the tumor is just in one hemisphere, such as the right or the left. Similarly, the image is spinning clockwise by an angle θ around the pixel in the center. This is achieved with the assistance of an appropriate interpolation that is tailored to fit the initial dimensions of the image. The rotational operation can be symbolized by the symbol τ, which is given in Equation (1).

(1) $$\tau =\left(\begin{array}{cc} cos\theta & -sin\theta \\ sin\theta & cos\theta \end{array}\right)$$

### 4.2. Segmentation

In the following step of the brain image segmentation procedure, an adaptive Canny Mayfly algorithm (ACMA)-based edge detection approach is utilized in order to locate the edges of the brain images. The image edge information, which can identify the target contour, relative placement within the target region, and other significant information, is one of the most important aspects of an image. Other key parts of an image include the center of the image and the pixels that make up the background. Edge detection is one of the most important processes in image processing since it greatly impacts image interpretation. The parameters of the typical Canny edge detection algorithm are chosen because the image quality is determined by elements such as noise and light during the process of image acquisition and because the spatial contrast of images in a large view field fluctuates. Researchers are unable to adapt the edge detection procedure to accommodate the diverse circumstances.

#### Canny Algorithm

The Canny algorithm (CA) is the method that is utilized in the medical industry more frequently than any other for edge detection [31]. Without any previous information, the location of the edges is determined here. Before the edge identification process begins, a smoothing technique called the Gaussian function is applied. In this case, the Gaussian noise has been removed, and the image’s resolution has been altered in such a way that it is now easily recognized. The Laplacian of Gaussian (LoG) filter is used first in the CA, which highlights the region with the most rapid-intensity transition. The supplied image is split in half using the LoG value as the dividing line. The value of the initial picture, which is referred to as the IoI (image of interest), is typically comparable to or even greater than the value of the LoG. In this case, the second image is not taken into consideration. After applying the LoG filter to the image, the following step is to smooth out the appearance of the image. In this case, a kernel is utilized to smooth out the image by drawing attention to the image’s edges. The kernel function is represented by Equation (2).

(2) $$K=\left[\begin{array}{ccc} 1 & 1 & 1\\ 1 & \beta & 1\\ 1 & 1 & 1 \end{array}\right]$$

In this case, β has a value of 2. When the parameter’s value is increased, further information can be received. However, the addition of more details does not always result in a clearer picture. As a result, the value of β was set as 2. After this stage of smoothing the image, the CA will look for the margins of the image where there is a significant amount of fluctuation in the intensity of the grey level. Image gradients are used to zero in on these regions. The maximum neighborhood pixels can be used to determine the gradient’s direction as well as its magnitude. Using the kernels, Equations (3) and (4) can determine the direction of the gradient.

(3) $$K\left(P_{x}\right)=\left[\begin{array}{ccc} -1 & 0 & 1\\ -2 & 0 & 2\\ -1 & 0 & 1 \end{array}\right]$$

(4) $$K\left(P_{y}\right)=\left[\begin{array}{ccc} 1 & 2 & 1\\ 0 & 0 & 0\\ -1 & -2 & -1\; \end{array}\right]$$

where $K\left(P_{x}\right)$ denotes the gradient of the kernel in the x direction and $K\left(P_{y}\right)$ denotes the gradient of the kernel in the y direction. The magnitude of the gradient can be found by solving Equation (5).

(5) $$\left|P\right|=\sqrt{\left(P_{x}^{2}+P_{y}^{2}\right)}$$

The direction of the edge α is calculated as given in Equation (6).

(6) $$\alpha =tan\frac{\left|P_{x}\right|}{\left|P_{y}\right|}$$

This expression is used to bring sharpness back to the edges of an image that has previously been blurry. Alternatively, it can sometimes expand beyond the boundaries of the image. Then, the edges cannot be calculated with complete accuracy. In this case, the Mayfly (MF) algorithm is used to optimize the edges’ orientation as well as the magnitude of the gradient [32]. The social behavior of MF served as the inspiration for the development of this program, particularly their mating procedure. The Mayfly algorithm allows for the values of $P_{x}$ and $P_{y}$ to be altered in various ways. In this process step, we update the equation by considering the behavior of male mayflies. If we define $d_{1}$ and $d_{2}$ as the distance coefficients and $a_{1}$ and $a_{2}$ as random coefficients in the range [−1, 1], then Equations (7) and (8) will give us the changed values of $P_{x}$ and $P_{y}$.

(7) $$P_{x}=P_{xi}+d_{1}\times a_{1}$$

(8) $$P_{y}=P_{yi}+d_{2}\times a_{2}$$

where $P_{xi}$ and $P_{yi}$ are shorthand notations for the beginning points of the gradients in the x and y directions, respectively. $d_{1}$ and $d_{2}$ are the symbols that are used to indicate the distance coefficients. Equations (7) and (8) show modified versions of the equations that were generated by the Mayfly method. When we plug these numbers into Equation (7), we get Equation (9).

(9) $$\alpha =tan\frac{\left|P_{xi}+d_{1}\times a_{1}\right|}{\left|P_{yi}+d_{2}\times a_{2}\right|}$$

**Figure 3:**
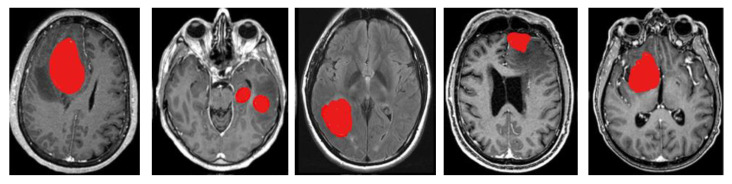
Segmentation results.

This is the final modified form of the Canny Mayfly equation. Next, the findings that were acquired from this step are sent on to the process of feature extraction. During this stage, the redundant information is removed so that the classification results can be improved. The segmentation results are shown in Figure 3.

### 4.3. Feature Extraction (FE)

The spatial grey-level dependence matrix approach, often known as SGLDM, is used to complete the FE process [33]. The primary objective of the SGLDM algorithm is to get rid of mathematical texture features in the second order so that the detection process may be made more accurate. Considered in this context is the joint conditional probability density function of the second order, which can be characterized by the notation R(i,j|v,μ). The value of μ is either 0, 45, 90, or 135 degrees.

Each R(i,j|v,μ) denotes a probability matrix, and it is arranged following the direction v and the distance of inter samples *v*. In this case, *i* and *j* stand for the various shades of grey. It is possible to indicate the predicted values for the PDF (probability density functions), as mentioned in Equation (10).

(10) $$\varphi \left(v,\mu \right)=R(i,j|v,\mu ),\;0<i,j\leq X_{g}$$

The value $X_{g}$ denotes the amount of grey that is the brightest possible. For a certain distance *v* = 1,2, we may extract the grey-level co-occurrence matrices, each of which has a unique value μ for the variable. The feature set is constructed using the ROI as the foundation, with selected texture characteristics from each ROI contributing to its construction.

### 4.4. Feature Selection

The FS procedure is carried out with the assistance of an altered version of the chimp optimization algorithm known as MChO [34]. Within a chimp colony, there are four distinct groups of chimps: the drivers, the barrier, the chasers, and the attackers. They each have their own set of skills, but the variety of these skills is what makes for a thrilling hunt. In most cases, the prey will be hunted down during exploitation and discovery procedures. The statistical model of the prey moving and being chased is expressed by Equations (11) and (12).

(11) $$D_{r}=\left|a_{1}\times S_{prey}\left(t\right)-a_{2}\times S_{chimp}\left(t\right)\right|$$

(12) $$S_{chimp}\left(t+1\right)=S_{prey}\left(t\right)-a_{3}\times a_{1}$$

where the letter *t* denotes the current iteration number, and the numbers $a_{1},\;\;a_{2},$ and $a_{3}$ refer to the coefficient vectors. The notations denote the location vectors of the chimp and its prey by $S_{chimp}$ and $S_{prey},$ respectively.

(13) $$a_{3}=2\times f\times r_{1}-f$$

(14) $$a_{1}=2\times r_{2}\;$$

Through the process of iteration, *f*′s value can be brought down from 2.5 to 0 in a non-linear way. The range of random vectors that can be found between 0 and 1 is denoted by the symbols $r_{1}$ and $r_{2}$. Equations (13) and (14) can be changed by employing sine and cosine functions in order to reach an optimal solution and also to reduce the amount of computing complexity involved, as shown in Equations (15) and (16).

(15) $$a_{3}=2\times f\;sin\left(r_{1}\right)\;-\;f$$

(16) $$a_{1}=2\times cos\left(r_{2}\right)$$

Consequently, the final enhanced chimp optimized equation for the feature selection process can be found by inserting Equations (15) and (16) in (11) and (12), respectively.

(17) $$D_{r}=\left|\left(2\times cos\left(r_{2}\right)-f\right)\times S_{prey}\left(t\right)-a_{2}\times S_{chimp}\left(t\right)\right|\;$$

(18) $$S_{chimp}\left(t+1\right)=S_{prey}\left(t\right)-\left[2\times f\times cos\;\left(r_{2}\right)\left(2\times sin\;\left(r_{1}\right)-1\right)\right]$$

The value of the parameter $a_{2}$ can take on any value between 0 and 1. The range [0, 1] is also utilized for the selection of the random vectors $r_{1}$ and $r_{2}$. Equations (17) and (18) are the modified equations of chimp optimization, and they are employed in the feature selection process to choose the helpful features from the dataset. These equations reflect the enhanced equations of chimp optimization. The results that are collected from this stage are then sent on to the classification procedure so that the MRI tumors can be classified appropriately.

### 4.5. Feature Classification

In the process of classification, a well-known deep residual network ResNet-152 is utilized as the pre-trained network in DCNN. This network is responsible for handling the vanishing gradient problem [34]. To continue the classification process, the output of ResNet-152 is sent to the softmax classifier (SMC) [35]. The following section covers the procedure of identifying and categorizing features. Convolutional layers (CL), downsampling layers (DSL), and fully connected layers are some of the most common types of layers that make up a deep convolutional neural network (DCNN) (FCL). The network depth of DL models plays a highly significant part in the process of achieving improved classification results. As a result, layers have been added to a CNN in order to improve its depth. After a specific value, when the CNN deepens, the accuracy of the network begins a slow but steady decline from that point on. A mapping function has been included in ResNet-152 in an effort to reduce the impact of the degradation problem. The expression of the mapping function is presented in Equation (19).

(19) W(x)=K(x)+x

*W*(*x*) is the mapping function constructed using a feed-forward neural network in conjunction with SC. The letter x denotes the input to the network. In most cases, SC is the identity mapping, which is the result of bypassing some layers directly, and $K\left(x,G_{i}\right)$ is the representation of the residual mapping function. The expression is represented by Equation (20).

(20) $$Z=K\left(x,G_{i}\right)+x$$

In the convolutional layers of the ResNet model, 3 × 3 filtering is utilized, and the downsampling operation with a stride of two is carried out. The classification results are produced by using a softmax layer in conjunction with global average pooling. After shortcut connections have been inserted, the ResNet is constructed. A dropout that is adaptive is used to represent the global average pooling in this example. The dropping indicates overfitting of half of the activations present in each hidden layer, which is accomplished with the dropout. An adaptive function can be used, as shown by Equation (21), to improve the performance of dropouts here.

(21) $$u=\frac{1}{n}\sum_{i=1}^{n}z\;log\;S_{i}+\left(1-z\right)\;log\;(1-S_{i})\;$$

where *n* is the number of training samples, *u* is the loss function, and $S_{i}$ is the output of the SMC. The SMC is a type of generalized logistic regression that may be used in many classes. It is a type of NN that is constructed in such a way that the activation function of the output layer guarantees that the outputs are in the range [0, 1] and always equal to one. The results of the SMC are shown in Equation (22).

(22) $$S_{i}=\frac{e^{l_{k}}}{\sum_{j=1}^{m}e^{y_{j}}\;},\;k=1,\cdots ,m;\;y=y_{1},\cdots ,\;y_{m}$$

In this case, the result of the softmax layer is expressed. $l_{k}$ is a component of the input vector, and *l*, *m* is the total number of neurons that are found in the output layer. The proposed method utilizes 152 convolutional layers (CLs), 10 adaptive dropout layers (ADLs), and a softmax classifier. The details of layers in ResNet-152 are listed in Table 1. 

**Figure 4:**
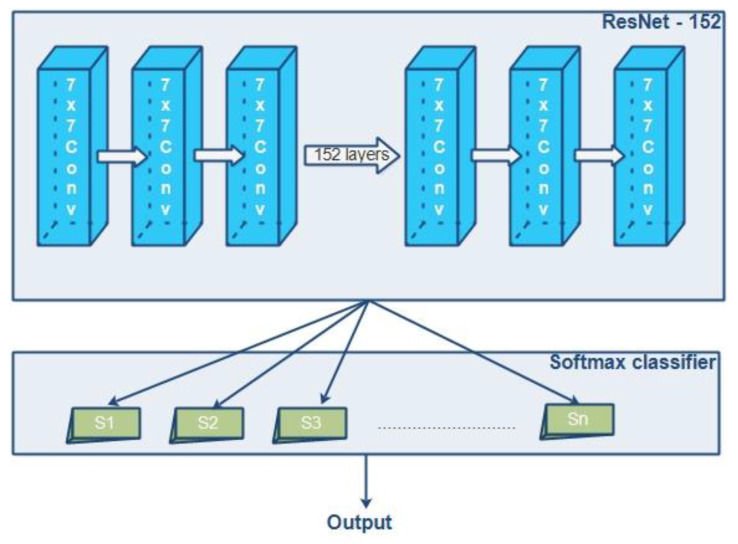
Architectural diagram of ResNet-152 and softmax classifier.

Figure 4 demonstrates the architecture that was utilized in the process. The model that is proposed is capable of accurately identifying many kinds of brain tumors, such as meningiomas, gliomas, and pituitary tumors. Multiple layers with 152 convolution filters can perform the learning process in a deep manner. This can ensure high accuracy in classification.

## 5. Results Analysis

Python was used as the platform to create an executable version of the proposed algorithm. In this methodology, the Figshare database is utilized to conduct experiments. The success of this strategy depends on a few different statistical criteria, namely classification accuracy, precision, and recall. These metrics are examined to demonstrate the proposed method’s effectiveness. The findings are then examined, and the classification methods used in the past are considered for comparison. This section describes the experimental results of the proposed methodology utilizing brain MR images collected from publicly available sources.

### 5.1. Dataset Description

The performance of the proposed methods is evaluated with different datasets such as Figshare [36], BRATS 2019 [37], and MICCAI BRATS BRATS 2019 [38]. This combined database primarily contains meningiomas, pituitary tumors, and gliomas of various tumors. 

### 5.2. Evaluation Parameters

With the use of statistical measurements such as sensitivity, specificity, and classification accuracy, it is possible to evaluate the overall performance of the method that has been proposed. The expressions of accuracy, precision, and recall are denoted by Equations (23)–(25), respectively, which are presented below. Equation (23) determines how accurate a value is by determining how near it is to the actual value.

(23) $$Accuracy=\frac{T_{P}+T_{N}}{T_{P}+T_{N}+F_{P}+F_{N}}$$

The ratio of the number of positive samples that are categorized compared to the total number of samples is the definition of precision. It is determined by Equation (24).

(24) $$Precision=\frac{T_{P}}{T_{P}+F_{P}}$$

Equation (25) determines the recall value, which is the ratio of samples categorized as positive to the total number of positive samples.

(25) $$Recall=\frac{T_{P}}{T_{P}+F_{N}}$$

The true negative and true positive values are indicated in Equations (22)–(24) with $T_{N}$ and $T_{P},$ respectively. Meanwhile, the notations $F_{N}$ and $F_{P}$ denote the false negative and false positive values, respectively.

### 5.3. Results and Discussion

The proposed method is analyzed by plotting its precision, accuracy, and recall on bar graphs to determine its overall effectiveness. Additionally, a ROC and confusion matrix are utilized to assess the performance. This methodology’s accuracy, precision, and recall are all examined. The data make it evident that the approach under consideration has an accuracy of around 98.85%, while its precision is approximately 96.81%. In addition, the recall performance is above average, coming in at approximately 97.64%.

**Figure 5:**
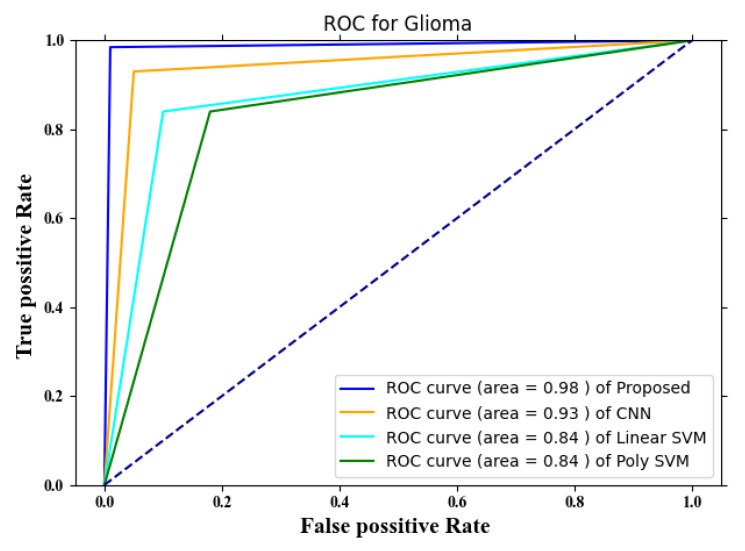
ROC for glioma tumors.

In this part of the article, the performance of the brain tumor classification method is evaluated compared to many other currently used methodologies. This study evaluates its performance based on the ROC and compares it with three existing approaches: CNN, linear SVM, and poly SVM [39]. A good indicator for determining the efficacy of a classification model is the area under the curve (ROC). The closer a curve is to the top left corner, the greater the accuracy of the outcome. The ROC for glioma tumors is shown in Figure 5.

**Figure 6:**
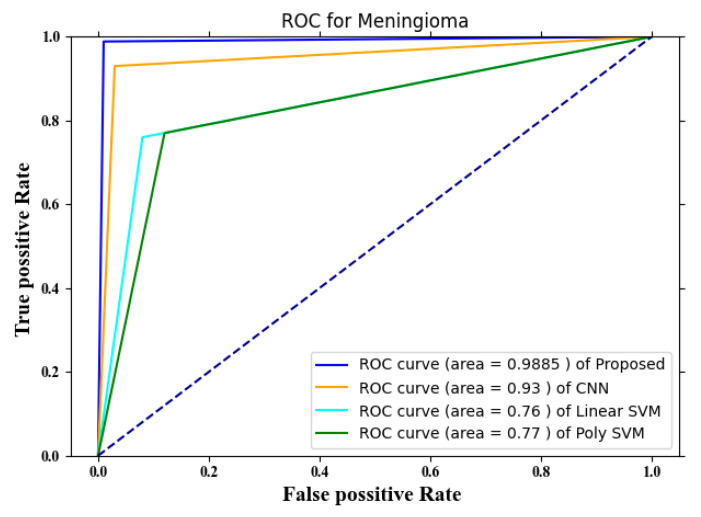
ROC for meningioma.

The area under the ROC for the proposed method of classifying brain tumors is depicted in Figure 3, which is 0.98. That indicates that the performance of the proposed technique demonstrates a superior performance for the classification of glioma compared to the performance of the other existing methods such as poly SVM, CNN, and linear SVM. The performances of poly and linear SVM are significantly worse than those of the other approaches. The ROC curve for meningioma is shown in Figure 6.

The TPR and the FPR are plotted against one another to get this graph. The image depicts four different methodologies: the proposed approach, CNN, poly SVM, and linear SVM. The proposed approach has a ROC for meningioma of 0.9885, which is higher than any of the other currently used methods. The area under the curve for CNN is 0.93, whereas the linear and polynomial SVMs have 0.76 and 0.77, respectively.

**Figure 7:**
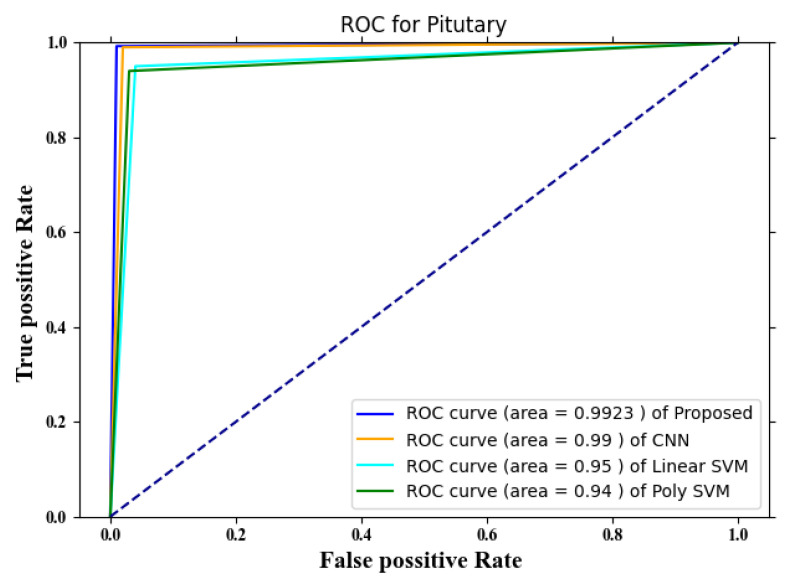
ROC for pituitary tumors.

The ROC of the pituitary gland is shown in Figure 7. According to the figure, the performance of the proposed approach is superior to those of the other three currently used methods. The ROC values that were calculated for SVM and poly SVM came in as 0.95 and 0.94, respectively. The graph makes it quite evident that the strategy that has been proposed yields superior results, with a ROC of approximately 0.9923. 

**Figure 8:**
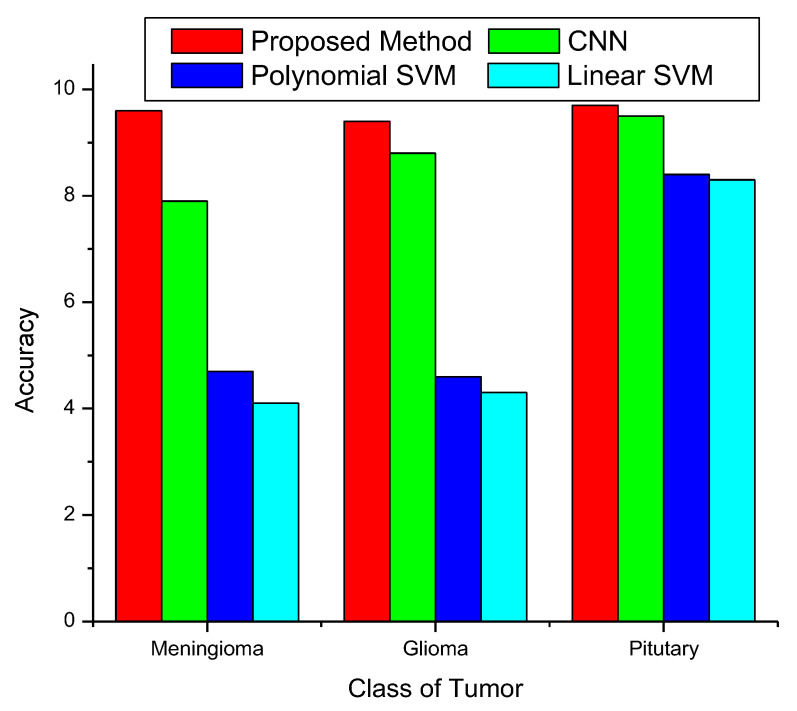
Accuracy of classification method.

Figure 8 presents an accuracy comparison graph. The first bar in the graph shows the proposed procedure’s accuracy. A comparison is made between the accuracy of the proposed approach and CNN, poly SVM, and linear SVM. The accuracy of this strategy can be summarized as 98.85%. The chart makes it abundantly evident that the overall accuracies of the SVM methods are subpar compared to those of the proposed and CNN approaches. Furthermore, the performance of the CNN approach is shown to be inferior to that of the proposed method. Overall, the accuracy when diagnosing meningiomas, gliomas, and pituitary tumors is superior to that of the CNN and SVM approaches. 

**Figure 9:**
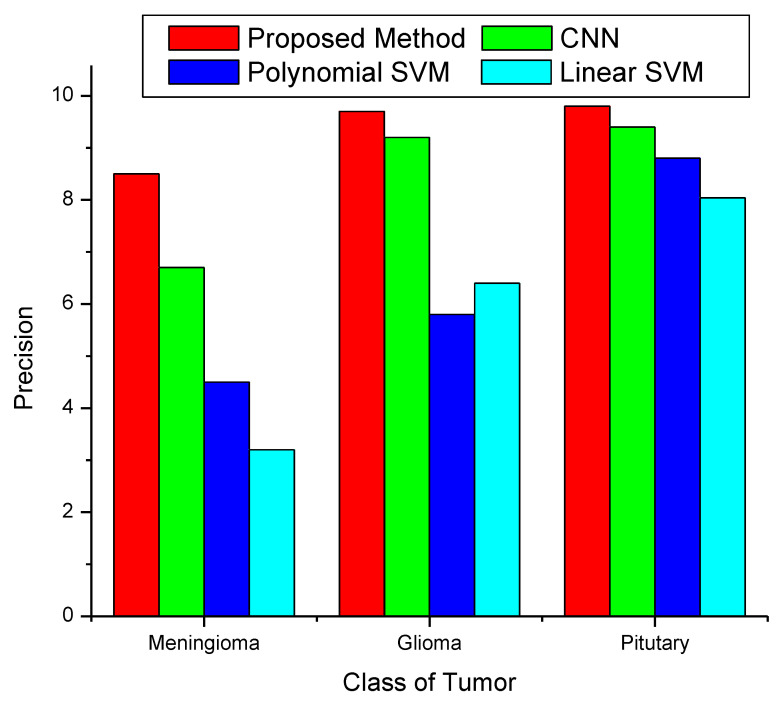
Precision of classification method.

Figure 9 illustrates a comparison of the precision of the proposed method with those of three other currently used methods. The graph makes it evident that the precision for all the classes, that is, meningioma, glioma, and pituitary tumors, is greater than for the CNN and SVM algorithms. The precision of the proposed approach for meningioma is around 93%, for glioma is approximately 99%, and for pituitary is 99.35%. 

**Figure 10:**
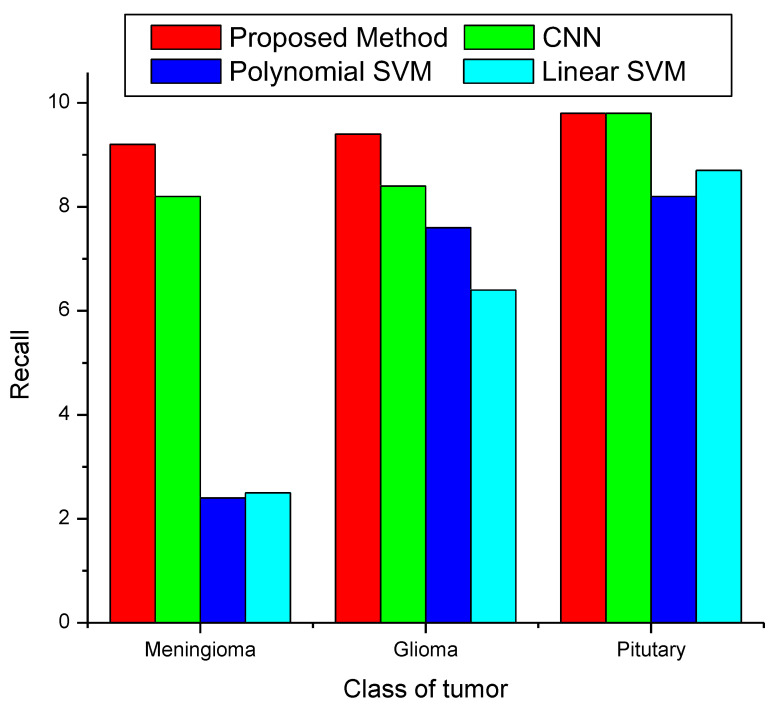
Recall classification.

A comparison of the recall of the proposed approach with three methods already in use is shown in Figure 10. Meningiomas have a recall value of 97%, gliomas 98%, and pituitary tumors 99% when using the proposed method. The graph makes it evident that the proposed method has a superior performance in recall compared to the other three strategies that are already in use. 

**Figure 11:**
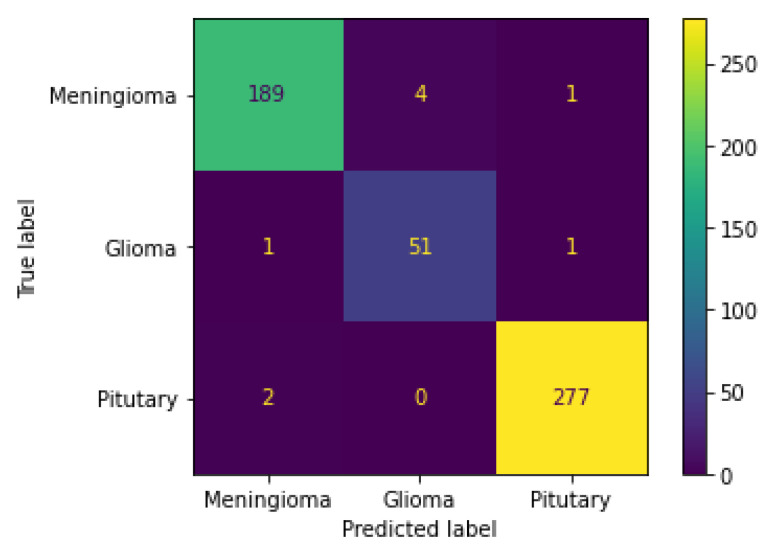
Confusion matrix.

Figure 11 illustrates the performance results in a confusion matrix and provides a summary of the output of the classifier. The two-dimensional layout includes both existing and anticipated occurrences. Instances that really occurred are listed in the matrix’s columns, while those anticipated are shown in the rows. Since it possesses a near-diagonal matrix, the deep convolutional neural network (DCNN) trained using ResNet-152 is the optimal choice for supplied classifiers. Table 2 compares the efficacy when using multiple classifications for brain tumors to those of several other methods currently in use.

**Figure 12:**
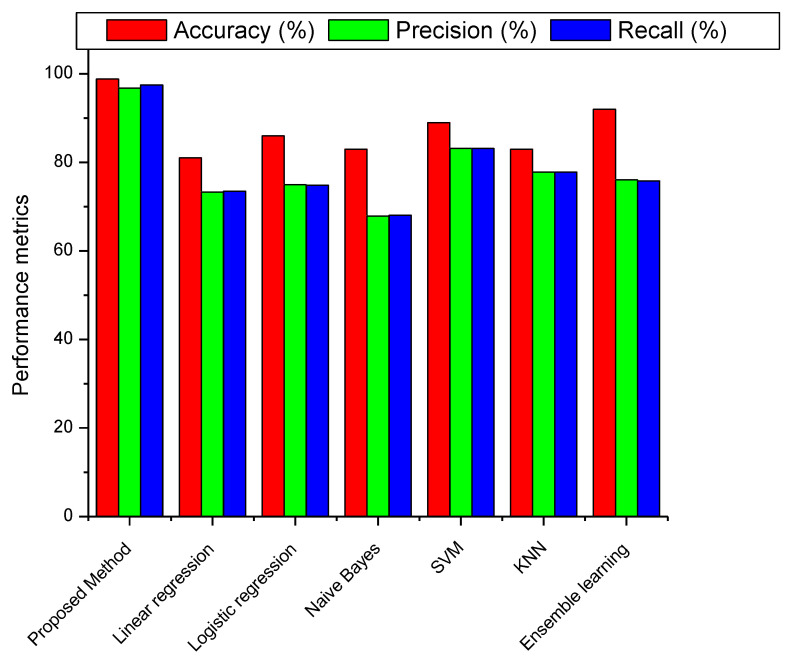
Comparison of performance metrics with currently used methods.

A performance comparison of the proposed multiclass brain tumor classification approach with some of the existing methods is shown in Figure 12. Linear discrimination (LD), logistic regression (LR), naive Bayes (NB), support vector machine (SVM), K closest neighbor (KNN), and ensemble learning (EL) are the existing methods taken into consideration here [40]. Accuracy, precision, and recall are the performance parameters considered during this stage of the review process. These parameters are expressed as percentages. The comparison chart makes it evident that the method that has been proposed outperforms every other method currently in use. The EL methodology has an accuracy of approximately 92%. In addition, the values obtained for precision and recall were 76.09% and 75.82%, respectively. In comparison, the accuracy of the SVM technique, its closest competitor, is approximately 89%. In a nutshell, the total performance reveals that the recommended technique performs better than the alternatives.

The performance levels when conducting classification on different datasets are summarized in Table 2. The results show that the proposed model outperforms the other methods.

The performance levels for segmentation with and without dimensionality reduction are compared on the three datasets in Table 3.

## 6. Conclusions

By applying a ResNet-152-based (DCNN) approach, this study achieves accurate multiclass classification of brain tumors. The involvement of ACMA improves the segmentation process’s effectiveness. Following the segmentation step, this approach moves on to the feature extraction phase, which takes the segmented image and extracts the features from it. The retrieved features take up huge space, which makes the classification system challenging to understand. To circumvent this problem, this method implements feature selection based on optimization. Finally, ResNet-152 and the softmax classifier using the chosen features flawlessly achieve the classification of brain tumors. We set out to create a powerful new deep learning network-based automatic classification system to classify the various forms of brain tumors. The use of a brand new adaptive Canny Mayfly algorithm for edge detection was implemented. We succeeded in recognizing malignancies such as meningioma, glioma, and pituitary tumors using the newly developed deep learning network structure of ResNet-152 as a pre-trained model in the deep convolutional neural network. A technique called data augmentation was used to enhance the large training dataset. With the use of a modified version of the chimp optimization technique, the superfluous features were eliminated. This improved the precision of classification. Accuracy, specificity, and sensitivity were the metrics reviewed and analyzed in the study. The proposed method has a precision rating of 96.81% and an accuracy rating of 98.85% overall. In addition to that, the recall value of the proposed strategy is 97.64%. According to the investigation’s findings, the proposed method demonstrates a superior performance compared to the existing methodologies. The work will be expanded by using numerous datasets to achieve superior evaluation findings in the future.

## Figures and Tables

**Table 1:** Details of ResNet-152 layers.

Name of the Layer	Stride	Input Size	Output Size	Additional Information
Convolution layer 1	2	7 × 7, 64	112 × 112	-
Convolution layer 2	2	$\left[\begin{array}{c} 1\times 1,\;64\\ 3\times 3,\;64\\ 1\times 1,\;256 \end{array}\right]\times 3$	56 × 56	3 × 3 maxpool
Convolution layer 3	1	$\left[\begin{array}{c} 1\times 1,\;128\\ 3\times 3,\;128\\ 1\times 1,\;512 \end{array}\right]\times 8$	28 × 28	-
Convolution layer 4	1	$\left[\begin{array}{c} 1\times 1,\;256\\ 3\times 3,\;256\\ 1\times 1,\;1024 \end{array}\right]\times 36$	14 × 14	-
Convolution layer 5	1	$\left[\begin{array}{c} 1\times 1,\;512\\ 3\times 3,\;52\\ 1x\times 1,\;2048 \end{array}\right]\times 3$	7 × 7	-
Average pooling	-	-	-	-
1000–fully connected layer	-	-	1 × 1	-

**Table 2:** Comparison on different datasets.

Dataset	Accuracy			Precision			Recall		
	Proposed Method	SVM	CNN	Proposed Method	SVM	CNN	Proposed Method	SVM	CNN
Figshare [36]	98	94	97	97	95	97	95	94	94
BRATS 2019 [37]	99	97	98	97	96	97	94	93	93
MICCAI BRATS [38]	99	98	97	96	97	96	96	94	94

**Table 3:** Comparison of segmentation accuracy with and without dimensionality reduction on different datasets.

Dataset	Accuracy	
	Without Dimensionality Reduction	With Dimensionality Reduction
Figshare [36]	98	97
BRATS 2019 [37]	99	98
MICCAI BRATS [38]	99	99

## Data Availability

Not applicable.
